# Characterization of 67 Confirmed Clustered Regularly Interspaced Short Palindromic Repeats Loci in 52 Strains of *Staphylococci*

**DOI:** 10.3389/fmicb.2021.736565

**Published:** 2021-10-22

**Authors:** Ying Wang, Tingting Mao, Yinxia Li, Wenwei Xiao, Xuan Liang, Guangcai Duan, Haiyan Yang

**Affiliations:** ^1^College of Public Health, Zhengzhou University, Zhengzhou, China; ^2^The First Affiliated Hospital, Sun Yat-sen University, Guangzhou, China

**Keywords:** *Staphylococcus aureus*, CRISPR-Cas, protospacer, crRNA, gene transfer

## Abstract

*Staphylococcus aureus* (*S. aureus*), which is one of the most important species of *Staphylococci*, poses a great threat to public health. Clustered regularly interspaced short palindromic repeats (CRISPR) and their CRISPR-associated proteins (Cas) are an adaptive immune platform to combat foreign mobile genetic elements (MGEs) such as plasmids and phages. The aim of this study is to describe the distribution and structure of CRISPR-Cas system in *S. aureus*, and to explore the relationship between CRISPR and horizontal gene transfer (HGT). Here, we analyzed 67 confirmed CRISPR loci and 15 companion Cas proteins in 52 strains of *Staphylococci* with bioinformatics methods. Comparing with the orphan CRISPR loci in *Staphylococci*, the strains harboring complete CRISPR-Cas systems contained multiple CRISPR loci, direct repeat sequences (DR) forming stable RNA secondary structures with lower minimum free energy (MFE), and variable spacers with detectable protospacers. In *S. aureus*, unlike the orphan CRISPRs away from *Staphylococcal* cassette chromosome *mec* (SCC*mec*), the complete CRISPR-Cas systems were in J1 region of SCC*mec*. In addition, we found a conserved motif 5′-TTCTCGT-3′ that may protect their downstream sequences from DNA interference. In general, orphan CRISPR locus in *S. aureus* differed greatly from the structural characteristics of the CRISPR-Cas system. Collectively, our results provided new insight into the diversity and characterization of the CRISPR-Cas system in *S. aureus*.

## Introduction

*Staphylococcus* is a genus of Gram-positive bacteria that can be found in the air, water, dust, and the skin and mucous membranes of humans and other organisms. Most of them are harmless and a few can cause diseases, for example, *Staphylococcus pseudintermedius*, *Staphylococcus schleiferi*, *Staphylococcus argenteus*, *Staphylococcus epidermidis*, and *Staphylococcus lugdunensis*. *Staphylococcus aureus* (*S. aureus*), as the most noticeable species in *Staphylococci*, is an important pathogen that can cause a series of diseases, including food poisoning, pneumonia, endocarditis, and sepsis, thus causing property losses and public health problems (Lowy, 1998; Hoffman et al., 2015; Vega and Dowzicky, 2017). The emergence and evolution of healthcare-associated methicillin-resistant *S. aureus* (HA-MRSA), community-associated MRSA (CA-MRSA), and livestock-associated MRSA (LA-MRSA) have greatly contributed to the pressure of human response to *S. aureus* infection (DeLeo et al., 2010; Gray et al., 2010; Aires-de-Sousa, 2017). Notably, in recent years, numerous studies have reported the detection of MRSA or multidrug-resistant *S. aureus* (MDRSA) in retail food worldwide (de Lencastre et al., 2017; Ge et al., 2017; Tang et al., 2017; Wang et al., 2017), indicating a shift to non-special locations. This is directly related to the popularization of antibiotics and the frequent horizontal transfer of mobile genetic elements (MGEs), such as phages, plasmids, transposons, and *Staphylococcal* cassette chromosomes (SCC) (Holden et al., 2013; Lindsay, 2014; Strommenger et al., 2014). It is obvious that these exogenous genes provide additional advantages for the survival of bacteria, but they also cause unavoidable problems, such as bacterial lysis caused by bacteriophage infection.

As prokaryotes evolved to meet challenges *in vivo* and *in vitro*, they developed an adaptive immune system called clustered regularly interspaced short palindromic repeats (CRISPR) and CRISPR-associated proteins (CRISPR-Cas) system to combat foreign MGEs, such as plasmids and phages, thus seeking the continuation of relative balance of species in continuous biological evolution. These systems consist of CRISPR loci, including highly conservative direct repeat sequences (DR) and spacer sequences derived from invading genes, and companion *cas* genes. According to the composition and structural characteristics of Cas protein complex, CRISPR-Cas systems are divided into 2 classes, 6 types, and 30 subtypes (Couvin et al., 2018; Makarova et al., 2020). Class 1 is defined by the presence of a multisubunit crRNA-effector complex, including type I, III, and IV, such as the effector module composed of Cas10, small subunit (SS) protein, Cas5, and severe paralogous Cas7 proteins in type III (Watson et al., 2021). Class 2 is defined by the presence of a single subunit crRNA-effector module, including type II, V, and VI, such as Cas9 in type II (Makarova et al., 2015). The unique signature proteins of these 6 types are Cas3 for type I, Cas9 for type II, Cas10 for type III, Csf1 for type IV, Cpf1 for type V, and Cas13 for VI (Makarova et al., 2011; Shmakov et al., 2015; Zetsche et al., 2015; Jung et al., 2016). At present, the subtypes reported in *S. aureus* are III-A and II-A (Golding et al., 2010; Ran et al., 2015; Aires-de-Sousa, 2017; Guan et al., 2017; Larsen et al., 2017). These type III-A CRISPR-Cas systems are either located in *Staphylococcal* cassette chromosome *mec* (SCC*mec*) or adjacent to SCC*mec*, supporting this hypothesis that the CRISPR-Cas systems can be transferred by MGE (Yang et al., 2015). Watson et al. (2018) proved bacteria could acquire an entire chromosomal CRISPR-Cas system through transduction.

Clustered regularly interspaced short palindromic repeats immunity is in 3 main stages: adaptation (spacer acquisition), expression (crRNAs formation), and interference (invading nucleic acids degradation). There are some slight differences among the various CRISPR-Cas types. The exogenous DNA sequence corresponding to spacer captured by the CRISPR-Cas system is called a protospacer (Moreb and Lynch, 2021). The main difference between a protospacer in invading nucleic acids and a spacer in CRISPR is the peripheral sequence. To distinguish self from non-self, type I and type II systems rely on 2–4 nt protospacer-adjacent motif (PAM), which is a short sequence adjacent to a protospacer and highly conserved that is not present in the host’s own CRISPR repeats (Wang et al., 2019). While, in type III and VI, typical PAM sequences were not found (Leenay and Beisel, 2017), they rely on the crRNA “tag,” an 8-nt sequence derived from the CRISPR repeats located at the 5’ flank of mature crRNA (Wang et al., 2019). PAM sequences play an important role in the acquisition of adaptive spacers and in the recognition and targeted cleavage of foreign DNA. Thus, phages can escape CRISPR attack by mutating PAM sequences (Collias and Beisel, 2021). The primed CRISPR adaptation (priming), as a pathway to spacer acquisition, was observed in type I systems, which resulted in spacer acquisition from location near the site of confirmed protospacers, indicating spacer acquisition is not random (Jackson et al., 2017). Furthermore, Nicholson et al. (2018) found evidence of a priming-like pathway in type II systems using bioinformatics methods. As opposed to type I and II systems, priming is not found in type III.

With the development of life sciences, the research of the CRISPR-Cas system has deepened. Nowadays, the CRISPR-Cas system is widely used in expression regulation, gene editing, nucleic acid detection, cell imaging, DNA assembly, and other fields (Ma et al., 2020; Ahmed et al., 2021; Ghaffari et al., 2021; Khajanchi and Saha, 2021; Luthra et al., 2021). Wang et al. (2020) developed a novel one-pot toolbox with precision and ultra-sensitivity platform for foodborne pathogen detection based on Cas12a/crRNA. Curti et al. (2020) demonstrated that Cas12a was able to detect DNA target sequences corresponding to carbapenemases resistance genes such as KPC, NDM, and OXA.

Bioinformatics analysis can help us understand CRISPR more comprehensively. *S. aureus* has SCC*mec*, which is obviously different from other bacteria, so the characteristics of the CRISPR-Cas system may be different from other systems. However, systematic bioinformatics analysis of *S. aureus* genome sequence structure is rarely reported. The main purpose of this study is to describe the distribution and structure of the CRISPR-Cas system in *S. aureus*, and to explore the relationship between CRISPR and horizontal gene transfer (HGT). We analyzed 67 confirmed CRISPR loci and 15 subtype III-A Cas proteins in 52 strains of *Staphylococci*. The structural characteristics and possible functions of CRISPR in *Staphylococci* were investigated by bioinformatics methods. We also analyzed the relationships between CRISPR loci and multilocus sequence typing (MLST), *S. aureus* protein A gene (*spa*), and SCC*mec*. The bioinformatics tools were used to explore the RNA secondary structures of DRs, the protospacers and potential PAM of spacers, and the phylogenetic tree of Cas proteins. We further studied the composition of spacers from each CRISPR locus and III-A Cas proteins.

## Materials and Methods

### Data Source

We downloaded 325 complete genomes of *Staphylococci* from the National Center for Biotechnology Information (NCBI) nucleotide database^1^ with default parameters (updated before August 30, 2018). The confirmed CRISPR loci were searched by the CRISPR Finder^2^ and *cas* genes by the CRISPRCas Finder^3^.

### Analysis Methods

The confirmed CRIPSR loci were found in 42 strains of *S. aureus* and the complete CRISPR-Cas system was identified in 15 strains of *Staphylococci* by the CRISPR Finder. To further verify the accuracy of the CRISPR array, CRISPRone was used for CRISPR array analysis of 15 strains of *Staphylococci* with type III-A system identified by the CRISPR Finder^4^ (Zhang and Ye, 2017). The phylogenetic tree of *cas* genes was performed by MAGE-X. The information about MLST, *spa*, and SCC*mec* of *S. aureus* was obtained from the Center for Genomic Epidemiology^5^. Consensus direct repeat sequences (CDRs) of CRISPR loci were recruited by removing redundance and not forming RNA secondary structures. The RNA secondary structures and minimum free energy (MFE) of CDRs were predicted by the RNA fold Web server, the output option is set to default^6^. Spacers in the CRISPR loci flanking the complete Cas cluster were analyzed. The difference between two spacer sequences below 5% is a homologous sequence by BLAST^7^. *CRISPRTarget* was used to identify protospacers in plasmids and phage database and to learn about the products encoded by the genes in which they reside^8^. The bases at both ends of the protospacer were collected according to the number of bases in the hairpin rings of the RNA secondary structure of the repeated sequences.

## Results

### CRISPR Loci of S. *aureus* in the CRISPR Database

**FIGURE 1:**
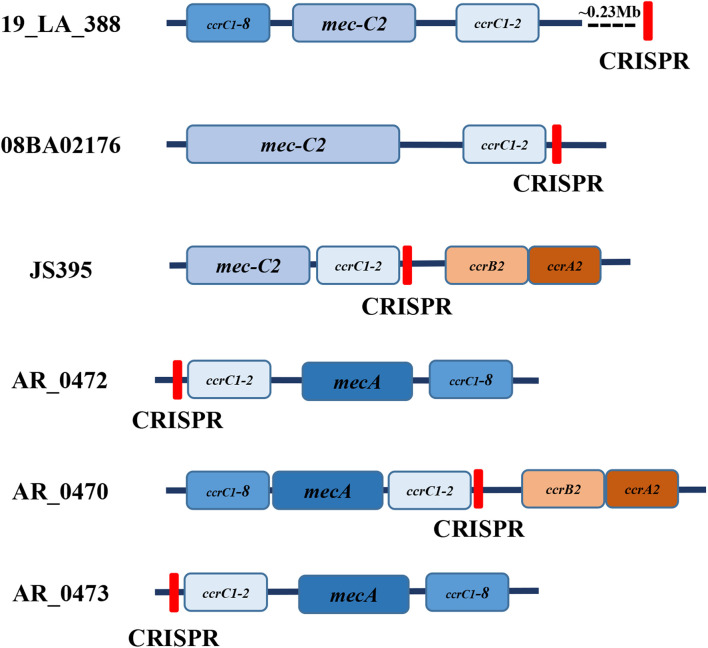
The position of CRISPR in MRSA. Strain 19_LA_388 is an example of strains carrying orphan CRISPR. The complete CRISPR-Cas systems in strain 08BA02176, JS395, AR_0472, AR_0470, and AR_0473 are in the J1 region.

There were 52 strains of *Staphylococci* detected to carry 67 confirmed CRISPR loci with 3 or 4 Cas3 proteins in 325 strains of *Staphylococci*, including 42 strains of *S. aureus* (Supplementary Tables 1, 2). There were 15 strains found to contain complete III-A Cas proteins by the CRISPR Finder and CRISPRone (Supplementary Table 3). They were *S. aureus* 08BA02176, *S. aureus* JS395, *S. aureus* AR_0472, *S. aureus* AR_0470, *S. aureus* AR_0473, *S. pseudintermedius* 063228, *S. schleiferi* TSCC54, *S. equorum* KS1039, *S. argenteus* MSHR1132, *S. argenteus* XNO62, *S. argenteus* XNO106, *S. epidermidis* FDAARGOS_153, *S. lugdunensis* N920143, *S. lugdunensis* HKU09-01, and *S. epidermidis* RP62A (with two questionable CRISPR loci). As shown in Supplementary Table 2, 41 strains of *Staphylococci* contained only 1 CRISPR locus, 6 strains contained 2 CRISPR loci, and the remaining contained 3 loci. Also, 19 ST, 20 *spa* types, and 8 SCC*mec* types were found among the 42 strains of *S. aureus*, of which the most representative was ST398-t034-Vc. Strain AR_0472, AR_0470, AR_0473, AR466, and AR_0471 cannot be assigned to the SCC*mec* type because of the lack of SCC*mec* components. In addition, as to the position of CRISPRs in *S. aureus*, unlike the orphan CRISPRs away from SCC*mec*, the complete CRISPR-Cas systems were in the J1 region (Figure 1).

### RNA Secondary Structure of Repeat Sequences

**FIGURE 2:**
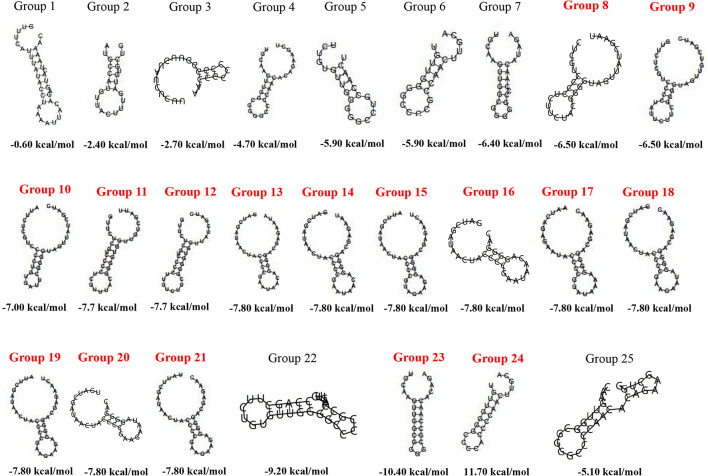
The RNA secondary structures and the MFE of the 25 group of CDRs. The CDRs from the CRISPR-Cas system are marked in red.

Repeat sequences always maintain a high degree of similarity and even are identical in one CRISPR locus. Therefore, the CDR of each CRISPR locus was chosen as the representative to predict the RNA secondary structure and MFE. A total of 25 CDRs were recruited by removing redundance and not forming RNA secondary structures from 67 confirmed CRISPR loci in 52 strains of *Staphylococci* (Supplementary Table 4). The length of CDRs was concentrated in 23–37 bp. Compared with CDRs from an orphan CRISPR array, CDRs from a complete CRISPR-Cas system were more likely to form stable RNA secondary structures with lower MFE (Figure 2). Notably, group 13–21 had different CDRs length, their MFE were all −7.8 kcal/mol, and the stem of their secondary structure was 4 “G-C” base pairs. The secondary structure of group 22 and group 25 was complicated with 3 rings and 2 stems. Comparing with other CDRs forming conservative dumbbell-shaped RNA secondary structures, the CDRs of these two groups were overlength and the bases forming the stem were distributed at one end of the sequences.

### The Homologous Analysis of Spacers

Among the 67 confirmed CRISPR loci, a total of 313 spacers were found. The difference between two spacer sequences below 5% was the same spacer sequence. Based on this principle, a total of 130 unique spacers were generated. In the process, we identified that the spacers in some CRISPR loci appeared to be highly homologous, all of which belonged to the strains harboring orphan CRISPR array. The length of spacers was concentrated in 19–53 bp. Since some DRs from a CRISPR locus could not form an RNA secondary structure, 127 spacers remained and then were employed as queries to search protospacers in phage and plasmid database by *CRISPRTarget*. Finally, 59 spacers hit 782 protospacers. These spacers belonged to the genomes carrying the complete CRISPR-Cas system and their length was concentrated in 29–43 bp. Furthermore, the provenance of the detected protospacers was examined. About 70% of protospacers mapped to hypothetical protein or unannotated genes. A clear provenance was shown in 239 protospacers of 31 spacers (Supplementary Table 5). These genes containing protospacers were DNA binding protein, encapsidation protein, terminase, transposase, and so on. A small fraction of spacers targeted open reading frames (ORF). Notably, two spacers targeted phi PVL-like protein in phage StauST398-2 and phage phi 13.

**FIGURE 3:**
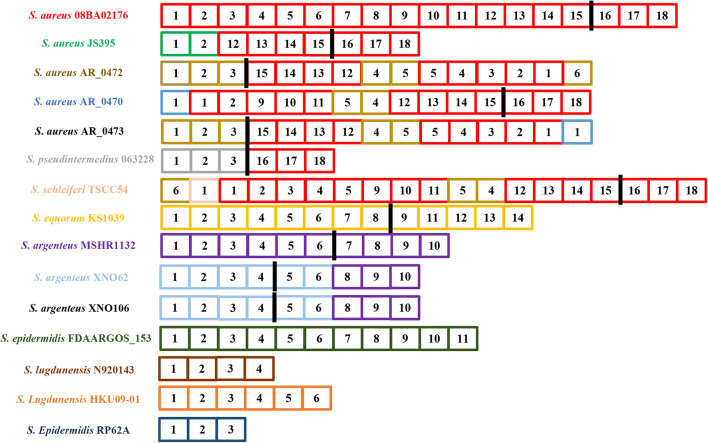
Graphic representation of spacers in the CRISPR loci flanking the complete Cas proteins. The strains shown in the figure all contain a complete type III-A CRISPR system. Repeats are not included. Two spacers are the same one when their diversity in DNA sequence is less than 5%. Each spacer consists of a small square, a number (continuously numbered in a strain), and a specific background color. The black rectangle represents the Cas cluster. The bacteria on the left are labeled as the color of the spacer as it originally appeared in its CRISPR locus. The strain labeled black indicates that there is no new spacer at its CRISPR locus.

By drawing the graphic representation of spacers in CRISPR loci flanking Cas proteins, we realized the spacers that make up the *S. aureus* 08BA02176 CRISPR array were also frequently found in other *S. aureus* CRISPR arrays, even in *S. pseudintermedius* 063228 and *S. schleiferi* TSCC54, especially the CRISPR loci composed of spacer 16–18 (Figure 3).

### Protospacer-Adjacent Motif and crRNA

**FIGURE 4:**
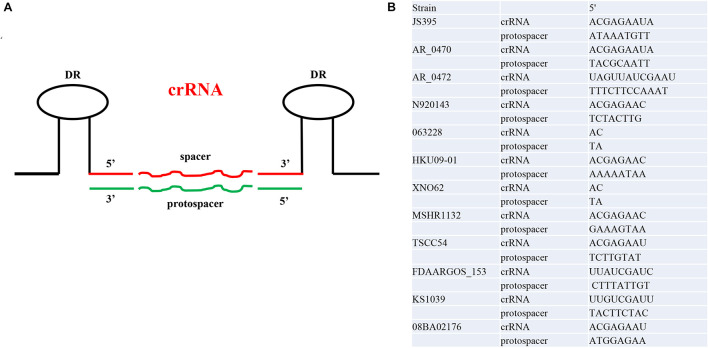
The schematic diagram and examples of the crRNA 5’ handle and protospacer 3’ end. **(A)** The schematic diagram of crRNA and protospacer. **(B)** Complementarity of crRNA 5’ handle and protospacer 3’ end. One *Staphylococci* has a representative.

In addition to exploring protospacer, *CRISPRTarget* is a rapid tool to identify potential PAM sequences. The portion of the crRNA consisting of DRs was presumed based on the secondary structure of the DRs. The length of crRNA 5′ handle was concentrated in 2–12 nt and crRNA was 40–62 nt. The nucleotides at both ends of the protospacers were extracted according to the number of nucleotides at the corresponding end of the crRNA. By removing redundance, a total of 176 PAMs were incorporated into the base pairing analysis. No complete base pairing between crRNA and PAM was found. We found that 51% (90/176) of crRNA 5′ handle contain the conserved motif 5′-ACGAGAA-3′ (shown in the Supplementary Materials). Therefore, we thought that the conserved motif 5′-ACGAGAA-3′ was widely distributed in crRNA 5′ handle. In *S. lugdunensis* N920143, the five base pairs at −8, −4, −3, −2, and −1 were the motifs with the most base pairs between crRNA and PAM (Figure 4).

### *cas* Cluster Near CRISPR Loci

**FIGURE 5:**
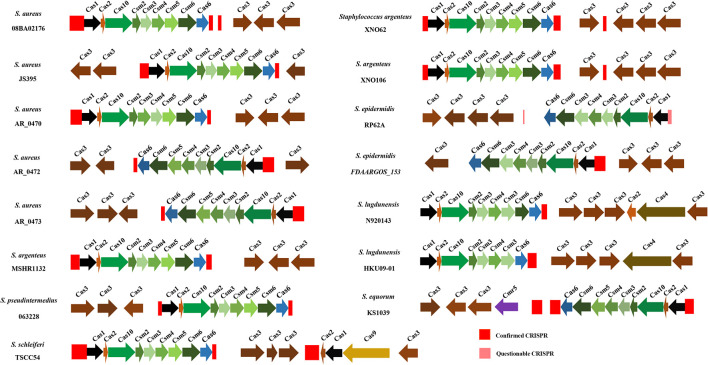
The distribution of CRISPR and *cas* genes in 15 *Staphylococci*. The red and pink rectangles represent confirmed CRISPRs and questionable CRISPRs, respectively.

In this study, 15 strains of *Staphylococci* were found carrying a complete CRISPR-Cas system and these *cas* gene clusters appeared to be highly homologous. As shown in Figure 5, the architecture of complete III-A Cas protein genes is *cas1*, *cas2*, *cas10*, *csm2*, *csm3*, *csm4*, *csm5*, *csm6*, and *cas6*. However, in *S. epidermidis* FDAARGOS_153, *S. lugdunensis* N920143, *S. lugdunensis* HKU09-01, and *S. epidermidis* RP62A, *csm5* has been replaced by *csm3*, whose length was the same as that of *csm5* in this position. Moreover, in *S. lugdunensis* HKU09-01, *csm*6 disappeared. A complete II CRISPR-Cas system was found in *S. schleiferi* TSCC54, presumably belonging to subtype IIC. There was no difference in the overall topology of phylogenetic trees among *cas1*, *cas2*, and complete *cas* genes, due to the highly homologous (Supplementary Figure 1).

## Discussion

As an adaptive prokaryotic immune system, the CRISPR-Cas system exists widely in nature. However, there are relatively few confirmed CRISPR loci in *Staphylococcus*. In this study, only 42 strains (12.92%) of *S. aureus* among 325 strains of *Staphylococci* carried confirmed CRISPR loci, far lower than other bacteria did (Watson et al., 2021). However, during the process of study, we found that there were several questionable CRISPR loci in each strain of *S. aureus*. We speculated that there were several reasons for this phenomenon. One is the lack of a unified discriminating standard, which leads to confusion in the confirmed and questionable CRISPR loci. For example, *S. epidermidis* RP62A is a model for Type III-A CRISPR-Cas systems (Makarova et al., 2015), but only two questionable CRISPR loci were found by CRISPR Finder. Another is no experimental evidence to prove whether these questionable CRISPR loci can play a role in the fighting against exogenous genes. Finally, it is possible to discover new CRISPR-Cas types (Nethery et al., 2021).

In this study, we analyzed the structures of 67 confirmed CRISPR loci in 52 strains of *Staphylococci*. Unlike the spacers in the orphan CRISPR loci, spacers in a complete CRISPR-Cas system showed high diversity in its CRISPR locus, consistent with the necessary for bacterial self-defense systems, which to some extent indicated that the acquisition of the new spacer was not affected by the pre-existing spacers. This was quite different from other subtypes in class 1, such as I-B, I-C, I-E, and I-F where the acquisition of the new spacer was often influenced by older spacers present in the same host (Nicholson et al., 2018). Some experimental studies have proved that this was not random, and pre-existing spacers were necessary in the acquisition process of new spacers (Li et al., 2014; Staals et al., 2016). By further analysis of these spacers, we found that the horizontal transfer and recombination of CRISPR loci within the same species were very frequent, like in *S. aureus* and *S. argenteus*. We also observed the same phenomena between different but closely related species, like among *S. aureus*, *S. pseudintermedius*, and *S. schleiferi*. Our previous study speculated this may be related to the SCC*mec* elements (Yang et al., 2015). We further observed that these CRISPRs were in the J1 region (Golding et al., 2010). Regions other than the *ccr* gene complex and *mec* gene complex are regarded as joining (J) regions. The J1 region often includes several ORFs and regulator genes (Lakhundi and Zhang, 2018). This suggests that these CRISPR loci are on SCC*mec*, and SCC*mec* can move from strain to strain, which may move along with SCC*mec* in horizontal transfer. Cao et al. (2016) analyzed six clinical isolates of *S. aureus* with subtype III-A CRISPR-Cas systems and found that four ST630 strains had similar CRISPR-Cas systems with that of ST398 *S. aureus* 08BA02176. Combined with our results, the link between MLST typing and CRISPR requires further studies.

Short palindromic sequences exist in direct repeats. Kunin et al. (2007) have shown that CRISPR repeats were structurally uneven, so the RNA secondary structures that may be formed during these direct repeats are studied. This structure can be combined with crRNA transcribed from the entire CRISPR sequence to form a bimodal structure to guide Cas protein to target the site (Yosef et al., 2012). Kunin et al. (2007) indicated that the stem-loop structure of some repeats may contribute to recognition-mediated contact between a gap-targeted exogenous RNA or DNA and a Cas-encoded protein, suggesting that the stability of RNA secondary structure may affect CRISPR function (He et al., 2012). Repeats from the CRISPR-Cas system were more likely to form stable RNA secondary structures with small MFE, while repeats from isolated CRISPR loci were more likely to form complex RNA secondary structures with unstable MFE (Wang et al., 2021). Our results showed that repeats from the CRISPR-Cas system were more likely to form stable RNA secondary structures with small MFE, while repeats from orphan CRISPR loci were more likely to form complex RNA secondary structures with unstable MFE. This further suggested that RNA secondary structure stability may enhance the function of CRISPR loci.

Spacers as genetic memories can show the history of past challenges from mobile genetic elements, such as bacteriophages and plasmids (Zhao et al., 2018). In this study, 59 spacers hit 782 protospacers. The vast majority of the protospacers originated from phage, indicating that those *Staphylococci* had been subjected to high frequency phage invasion and phage played an important role in the evolution of *Staphylococcus*. Levin (2010) have suggested that CRISPR immune bacteria and their phages are engaged in a co-evolutionary arms race, with host accumulation of spacers and phage accumulation point mutations. In recent years, this CRISPR-phage co-evolution model has been modified based on more refined models (Weissman et al., 2018; Common et al., 2019). Gophna et al. (2015) showed the inhibitory effect of CRISPR-Cas on HGT was undetectable on evolutionary timescales. Later, Watson et al. (2018) proved CRISPR-Cas-mediated phage resistance enhanced horizontal gene transfer by transduction. Notably, more protospacers have been detected with the development and popularity of next-generation sequencing technologies in recent years (Watson et al., 2018). Nevertheless, in a recent study, researchers found that protospacers were detectable for 1% to about 19% of the spacers and subtype III-A was 2% (Shmakov et al., 2017). More than half of the spacers targeted hypothetical protein, and for the rest, the enzymes and proteins necessary for bacterial growth and survival were the main targets.

Protospacer-adjacent motif plays a dual role: first, it initiates crRNA binding to the invading target sequence enabling interference, and second, prevents self-targeting (Gleditzsch et al., 2019; Collias and Beisel, 2021). The PAM allows the nuclease to discriminate between subsequent infection by the invader (non-self) from the invader-derived spacer sequence encoded in the CRISPR array (self) in type I, II, and V. Without the PAM requirement, CRISPR Cas systems would target their CRISPR arrays, leading to a potentially catastrophic autoimmune response (Collias and Beisel, 2021). Virtually, all CRISPR nucleases require a PAM in one form or another. However, the recognized PAM sequences are not shared by all Cas nucleases and instead vary widely, with different sequences, lengths, complexities, orientations, and distances from the target (Yosef et al., 2012). Therefore, the CRISPR Cas system will evolve and maintain PAM recognition as an absolute requirement for immune function under strict selection pressures (Collias and Beisel, 2021). Different CRISPR-Cas systems use diverse mechanisms to check the presence of the PAM sequence in the pre-spacer (Gleditzsch et al., 2019). In the *E. coli* subtype I-E system, this role is fulfilled by the Cas1 (Gleditzsch et al., 2019). Type III and VI evaluate the extent of complementarity between the flanking portions of the gRNA and target. Previous experimental researches suggested subtype III-A systems differentiated self and non-self sequences based on the complementarity between the crRNA 5′ handle and target 3′ terminal (Marraffini and Sontheimer, 2010; Samai et al., 2015). In the case of Type III and Type VI systems, limited evidence suggests that the PAM is located within the target RNA (Elmore et al., 2016). Because of this unique location, the PAM for these systems was renamed the RNA PAM (rPAM) or the protospacer-flanking sequence (PFS), respectively (Elmore et al., 2016). Both Deng et al. (2013) and Peng et al. (2015) observed DNA sequences downstream of pentanucleotide 5′-GAGAC-3′ and 5′-GAAAG-3′ could avoid DNA interference in subtype III-B Cmr systems. So, in this study, we focused on base pairing between the crRNA 5′ handle and target 3′ terminal and conserved motifs that were widely distributed in crRNA 5′ handle. We speculated that 5′-ACGAGAA-3′ seemed to be the conserved motif, then 5′-TTCTCGT-3′ was the protective motif, but further experimental studies are needed to verify our current findings. The potential impact of crRNA 5′ handle site mutations has also been studied in some studies. Cao et al. (2016) found that the first five nucleotides in the 5′ end did impact the CRISPR immunity in subtype III-A systems. Marraffini and Sontheimer (2010) found that protection of the CRISPR locus would disappear with at least two consecutive mismatches from position −4 to −2. Recently, Pyenson et al. (2017) indicated that PAM or seed sequences are not required for targeting in *S. epidermidis* type III-A CRISPR-Cas system. In subtype VI systems, researchers showed that repeat nucleotides at positions 1, 2, and 3 are the most important for the protective effects of a repeat sequence placed downstream of the protospacer (Meeske and Marraffini, 2018).

In type III systems, the adaptation module (Cas1 and Cas2) and repeat-associated mysterious protein (Cas6) are dispensable (Makarova et al., 2015). All strains of *Staphylococci* possessed these proteins in our study. As the best conserved Cas protein, Cas1 is mainly characterized by horizontal evolution in type III (Makarova et al., 2011; Vestergaard et al., 2014). Furthermore, the phylogeny of Cas1 and reverse transcriptase (RT) phylogeny suggested that the two proteins (or domains in fusion proteins) generally co-evolve (Silas et al., 2017). As for Cas2, other than the function in the adaptation module in *Legionella pneumophila*, it has been reported that Cas2 nuclease activity is critical for promoting infection in amoebae (Gunderson et al., 2015). Later, Dixit et al. (2016) found Cas2 possesses divalent metal-independent RNase activity in *Leptospira interrogans*. In type III systems, Cas6 is not a subunit of the effector complex. In a recent review, authors proposed a bivalent trapping and an unwinding mechanism for CRISPR-Cas6 to interact with the relaxed and the tight repeat RNA, respectively (Sefcikova et al., 2017). All type III systems encode the signature protein Cas10, SS protein, one Cas5 protein, and typically several paralogous Cas7 proteins (Makarova et al., 2015). During the interference stage, Cas-crRNA complexes function as sequence-specific nucleases to identify and degrade invading genetic material. Recent studies have shown that this is carried out by the Cas10 subunit in type III systems (Estrella et al., 2016; Kazlauskiene et al., 2016). By studying the interaction between a type III-A ribonucleoprotein complex and various RNA substrates, researchers found that the central role of the internal dynamics of CRISPR-Cas complexes in self- vs. non-self-discrimination and target specificity (Wang et al., 2019). Considering the case in our study, Csm5 was replaced by Csm3 in four strains of *Staphylococci* and one strain did not harbor Csm6. However, these CRISPR loci were active and no anti-CRISPR proteins, therefore a simpler *csm* complex could serve as the original function, or would CRISPR have a narrow target? For Csm6, as an RNase, it is essential for immunity when the target is located in late-expressed genes or contains mismatches to the spacer (Jiang et al., 2016).

In conclusion, this study primarily analyzed the structure of DRs and spacers, as well as the companion Cas proteins. The results showed that orphan CRISPR loci in *S. aureus* differed greatly from the structural characteristics of the CRISPR-Cas system. The strains harboring complete CRISPR-Cas systems contained multiple CRISPR loci, and whose DRs were likely to form the stable RNA secondary structures with lower MFE and spacers were variable with detectable protospacers. In addition, these CRISPRs were in the J1 region. The transfer of the CRISPR locus was frequent in *Staphylococcus*. Again, a conserved motif 5′-TTCTCGT-3′ that may protect their downstream sequences from DNA interference was found.

## Data Availability Statement

The original contributions presented in the study are included in the article/Supplementary Material, further inquiries can be directed to the corresponding author.

## Author Contributions

TM designed this study. YW and TM finished the experiments, collected the data, and wrote the manuscripts together. YW, YL, TM, WX, XL, GD, and HY revised the manuscript critically for important intellectual content. All authors read and approved the final manuscript.

## Conflict of Interest

The authors declare that the research was conducted in the absence of any commercial or financial relationships that could be construed as a potential conflict of interest.

## Publisher’s Note

All claims expressed in this article are solely those of the authors and do not necessarily represent those of their affiliated organizations, or those of the publisher, the editors and the reviewers. Any product that may be evaluated in this article, or claim that may be made by its manufacturer, is not guaranteed or endorsed by the publisher.

## Footnotes

1
http://www.ncbi.nlm.nih.gov/

2
https://crisprcas.i2bc.paris-saclay.fr/

3
https://crisprcas.i2bc.paris-saclay.fr/CrisprCasFinder/Index

4
http://omics.informatics.indiana.edu/CRISPRone

5
http://www.genomicepidemiology.org/

6
http://rna.tbi.univie.ac.at/cgi-bin/RNAWebSuite/RNAfold.cgi

7
https://blast.ncbi.nlm.nih.gov/Blast.cgi

8
http://bioanalysis.otago.ac.nz/CRISPRTarget/crispr_analysis.html#userconsent#
