# Supplementary material for: Characterization of 67 Confirmed Clustered Regularly Interspaced Short Palindromic Repeats Loci in 52 Strains of Staphylococci
Source: Front Microbiol. 2021 Oct 22;12:736565. doi: 10.3389/fmicb.2021.736565 (PMC8571024; doi:10.3389/fmicb.2021.736565)
Supplement: Supplementary file 1 [file Data_Sheet_1.docx]

Table S1. The accession number of 325 strains of *Staphylococci* in NCBI database

| No. | Strain | Accession number | No. | Strain | Accession number | No. | Strain | Accession number |
| --- | --- | --- | --- | --- | --- | --- | --- | --- |
| 1 | ST398 | AM990992 | 2 | 55-99-44 | CP024998 | 3 | NCTC8325 | CP000253 |
| 4 | JKD6159 | CP002114 | 5 | BB155 | LN854556 | 6 | RF122 | AJ938182 |
| 7 | LGA251 | FR821779 | 8 | AR466 | CP029080 | 9 | N315 | BA000018 |
| 10 | 08BA02176 | CP003808 | 11 | AR464 | CP029084 | 12 | Mu50 | BA000017 |
| 13 | CA-347 | CP006044 | 14 | PTDrAP2 | CP029172 | 15 | Mu3 | AP009324 |
| 16 | 104 | CP012409 | 17 | MOK063 | CP029629 | 18 | strNewman | AP009351 |
| 19 | BA01611 | CP019945 | 20 | AR_0472 | CP029649 | 21 | MW2 | BA000033 |
| 22 | JS395 | CP012756 | 23 | AR_0470 | CP029653 | 24 | MRSA252 | BX571856 |
| 25 | RIVM1295 | CP013616 | 26 | AR_0471 | CP029652 | 27 | MSSA476 | BX571857 |
| 28 | RIVM1607 | CP013619 | 29 | AR_0473 | CP029681 | 30 | COL | CP000046 |
| 31 | RIVM3897 | CP013621 | 32 | 5_3949 | LT992462 | 33 | USA300_FPR3757 | CP000255 |
| 34 | FDA209P | AP014942 | 35 | 8_LA_272 | LT992461 | 36 | JH9 | CP000703 |
| 37 | ST20130941 | CP012978 | 38 | 2_LA_86 | LT992463 | 39 | USA300_TCH1516 | CP000730 |
| 40 | E154 | CP013218 | 41 | 16_LA_309 | LT992467 | 42 | JH1 | CP000736 |
| 43 | 08S00974 | CP020019 | 44 | 12_LA_293 | LT992468 | 45 | ED98 | CP001781 |
| 46 | ATCC6538 | CP020020 | 47 | 17_LA_343 | LT992471 | 48 | 04-02981 | CP001844 |
| 49 | 293G | CP019591 | 50 | 10_5235 | LT992472 | 51 | TW20 | FN433596 |
| 52 | GD705 | CP019593 | 53 | 19_LA_388 | LT992474 | 54 | strJKD6008 | CP002120 |
| 55 | GD1677 | CP019595 | 56 | NCTC3761 | LS483314 | 57 | TCH60 | CP002110 |
| 58 | GD1539 | CP019594 | 59 | NCTC6136 | LS483311 | 60 | 55_2053 | CP002388 |
| 61 | T0131 | CP002643 | 62 | ATCC25923 | CP009361 | 63 | UTSWMRSA55 | CP013231 |
| 64 | ED133 | CP001996 | 65 | Gv69 | CP009681 | 66 | MCRF184 | CP014791 |
| 67 | M013 | CP003166 | 68 | FORC_001 | CP009554 | 69 | ST20130940 | CP012979 |
| 70 | 11819-97 | CP003194 | 71 | 29b_MRSA | CP010295 | 72 | ST20130942 | CP012976 |
| 73 | VC40 | CP003033 | 74 | 31b_MRSA | CP010296 | 75 | ST20130943 | CP012974 |
| 76 | ECT-R2 | FR714927 | 77 | 33b | CP010297 | 78 | ST20130938 | CP012972 |
| 79 | HO50960412 | HE681097 | 80 | 26b_MRSA | CP010298 | 81 | ST20130939 | CP012970 |
| 82 | 16035 | HE579065 | 83 | 25b_MRSA | CP010299 | 84 | RIVM6519 | CP015173 |
| 85 | Bmb9393 | CP005288 | 86 | 27b_MRSA | CP010300 | 87 | ZJ5499 | CP011685 |
| 88 | 6850 | CP006706 | 89 | DAR4145 | CP010526 | 90 | NCCP14558 | CP013953 |
| 91 | CN1 | CP003979 | 92 | ILRI_Eymole1_1 | LN626917 | 93 | NCCP14562 | CP013955 |
| 94 | SA957 | CP003603 | 95 | 10388 | HE579059 | 96 | V521 | CP013957 |
| 97 | SA40 | CP003604 | 98 | 10497 | HE579061 | 99 | 08-02119 | CP015645 |
| 100 | Z172 | CP006838 | 101 | 15532 | HE579063 | 102 | 08-02300 | CP015646 |
| 103 | USA300-ISMMS1 | CP007176 | 104 | 18341 | HE579069 | 105 | 1625.CO1 | CP016863 |
| 106 | 502A | CP007454 | 107 | 18412 | HE579071 | 108 | 2148.C01 | CP017094 |
| 109 | FDAARGOS_5 | CP007539 | 110 | FCFHV36 | CP011147 | 111 | FORC_027 | CP012692 |
| 112 | H-EMRSA-15 | CP007659 | 113 | M121 | CP007670 | 114 | FORC_026 | CP013132 |
| 115 | UA-S391_USA300 | CP007690 | 116 | CA15 | CP007674 | 117 | SA40TW | CP013182 |
| 118 | SA268 | CP006630 | 119 | RKI4 | CP011528 | 120 | HG001 | CP018205 |
| 121 | SJTUF_J27 | CP019117 | 122 | C2406 | CP019590 | 123 | K17 | CP020713 |
| 124 | NCTC13137 | LS483308 | 125 | GD5 | CP019592 | 126 | K18 | CP020714 |
| 127 | NCTC8726 | LS483302 | 128 | M92 | CP015447 | 129 | NZAK3 | LT009690 |
| 130 | USA300_SUR1 | CP009423 | 131 | FORC_039 | CP015817 | 132 | Sa_Newman_UoM | LT598688 |
| 133 | UCI62 | CP018766 | 134 | G477 | CP021905 | 135 | AUS0325 | LT615218 |
| 136 | UCI28 | CP018768 | 137 | G478 | CP021907 | 138 | isolateClinical | LT671859 |
| 139 | SR434 | CP019563 | 140 | ISU935 | CP017090 | 141 | O11 | CP024649 |
| 142 | USA300-SUR9 | CP014392 | 143 | FORC_045 | CP017115 | 144 | XN108 | CP007447 |
| 145 | USA300-SUR10 | CP014397 | 146 | FORC_040 | CP016398 | 147 | V605 | CP013959 |
| 148 | USA300-SUR11 | CP014402 | 149 | Newman_D2C | CP023391 | 150 | 2148N | CP016856 |
| 151 | USA300-SUR12 | CP014407 | 152 | NYU_Newman | CP023390 | 153 | 1969N | CP016861 |
| 154 | USA300-SUR13 | CP014409 | 155 | OC8 | AP017377 | 156 | NZ15MR0322 | LT699704 |
| 157 | USA300-SUR14 | CP014412 | 158 | JH4899 | AP014921 | 159 | TCH959 | CP026076 |
| 160 | USA300-SUR15 | CP014415 | 161 | FDAARGOS_412 | CP023500 | 162 | NRS137 | CP026080 |
| 163 | USA300-SUR16 | CP014420 | 164 | HZW450 | CP020741 | 165 | NRS149 | CP026063 |
| 166 | USA300-SUR17 | CP014423 | 167 | CFSAN007835 | CP017685 | 168 | NRS271 | CP026064 |
| 169 | USA300-SUR18 | CP014426 | 170 | CFSAN007847 | CP017684 | 171 | NRS153 | CP026067 |
| 172 | USA300-SUR19 | CP014429 | 173 | CFSAN007850 | CP017682 | 174 | NRS133 | CP026070 |
| 175 | USA300-SUR20 | CP014432 | 176 | CFSAN007851 | CP017680 | 177 | NCTC13395 | LS483316 |
| 178 | USA300-SUR21 | CP014435 | 179 | CFSAN007883 | CP017679 | 180 | NCTC11940 | LS483350 |
| 181 | USA300-SUR22 | CP014438 | 182 | CFSAN007894 | CP017677 | 183 | AR_0219 | CP029675 |
| 184 | USA300-SUR23 | CP014441 | 185 | NCTC5663 | LS483317 | 186 | AR_0216 | CP029678 |
| 187 | USA300-SUR24 | CP014444 | 188 | NCTC13140 | LS483319 | 189 | AR_0467 | CP029658 |
| 190 | JE2 | CP020619 | 191 | EDCC5458 | CP022290 | 192 | AR_0225 | CP029667 |
| 193 | NCTC9944 | LS483309 | 194 | EDCC5464 | CP022291 | 195 | AR_0469 | CP029655 |
| 196 | NCTC9752 | LS483310 | 197 | USA400-0051 | CP019574 | 198 | AR_0215 | CP029680 |
| 199 | CHU15-056 | CP021171 | 200 | 422 | CP022898 | 201 | WCH-SK2 | CP031537 |
| 202 | AR_0226 | CP029664 | 203 | 468 | CP022900 | 204 | 24117-WT | LT996891 |
| 205 | AR_0468 | CP029657 | 206 | 128 | CP022897 | 207 | 1549-WT | LT992434 |
| 208 | AR_0228 | CP029663 | 209 | 165 | CP022902 | 210 | 1549-REV | LT992436 |
| 211 | AR_0222 | CP029671 | 212 | 277 | CP022896 | 213 | 1549-SCV | LT992435 |
| 214 | AR_0220 | CP029673 | 215 | 61 | CP022893 | 216 | 24117-REV | LT996889 |
| 217 | ST2594 | CP027486 | 218 | 143 | CP022899 | 219 | 24117-SCV | LT996890 |
| 220 | CMRSA-3 | CP029685 | 221 | 466 | CP022901 | 222 | 1_1439 | LT992456 |
| 223 | CMRSA-6 | CP027788 | 224 | 191 | CP022894 | 225 | 3_LA_115 | LT992464 |
| 226 | AR_475 | CP030323 | 227 | 85 | CP022895 | 228 | 7_4623 | LT992458 |
| 229 | AR_474 | CP030326 | 230 | 54 | CP022892 | 231 | 9_LA_281 | LT992460 |
| 232 | E16SA093 | CP031131 | 233 | 45 | CP022718 | 234 | 6_LA_232 | LT992465 |
| 235 | F17SA003 | CP031130 | 236 | 164 | CP022910 | 237 | 4_LA_208 | LT992466 |
| 238 | FORC_061 | CP022607 | 239 | 78 | CP022682 | 240 | 13_LA_301 | LT992470 |
| 241 | FORC_062 | CP022582 | 242 | 135 | CP022720 | 243 | 14_5418 | LT992473 |
| 244 | NCTC8325-1 | LS483365 | 245 | 545 | CP022908 | 246 | 15_LA_305 | LT992469 |
| 247 | NCTC13277 | LS483484 | 248 | 546 | CP022906 | 249 | 20_LA_415 | LT992475 |
| 250 | 27 | CP022717 | 251 | 628 | CP022905 | 252 | 21_LA_436 | LT992476 |
| 253 | 187 | CP022903 | 254 | ISU926 | CP017091 | 255 | 22_LA_562 | LT992477 |
| 256 | 629 | CP022904 | 257 | NCTC10344 | LS483324 | 258 | NCTC13394 | LS483301 |
| 259 | NCTC7485 | LS483300 | 260 | NRS107 | CP026077 | 261 | NRS484 | CP026066 |
| 262 | O46 | CP025395 | 263 | NRS70 | CP026079 | 264 | NRS146 | CP026068 |
| 265 | 2395USA500 | CP007499 | 266 | NRS1 | CP026069 | 267 | CFSAN064037 | CP028165 |
| 268 | CA12 | CP007672 | 269 | NRS120 | CP026072 | 270 | CFSAN018750 | CP028189 |
| 271 | HUV05 | CP007676 | 272 | Mw2-1 | CP026073 | 273 | CIT | CP029031 |
| 274 | V2200 | CP007657 | 275 | HPV107 | CP026074 | 276 | CAR | CP029030 |
| 277 | USA300_2014.C01 | CP012119 | 278 | 16125 | HE579067 | 279 | AR461 | CP029087 |
| 280 | USA300_2014.C02 | CP012120 | 281 | 18583 | HE579073 | 282 | AR462 | CP029086 |
| 283 | HOU1444-VR | CP012593 | 284 | FDAARGOS_10 | CP026961 | 285 | FDAARGOS_1 | CP026968 |
| 286 | SA564 | CP010890 | 287 | FDAARGOS_15 | CP026960 | 288 | FDAARGOS_40 | CP026958 |
| 289 | GR2 | CP010402 | 290 | AR_0223 | CP029669 | 291 | K5 | CP020656 |
| 292 | XQ | CP013137 | 293 | 5118.N | CP016855 | 294 | FORC59 | CP020354 |
| 295 | MS4 | CP009828 | 296 | 3020.C01 | CP025495 | 297 | CFSAN007896 | CP020467 |
| 298 | NCTC13435 | LN831036 | 299 | CFSAN064038 | CP028163 | 300 | CC5 | CP021105 |
| 301 | Gv51 | CP012015 | 302 | CFSAN018749 | CP028190 | 303 | OXLIM | CP029032 |
| 304 | Be62 | CP012013 | 305 | MRSA107 | CP018629 | 306 | AR465 | CP029082 |
| 307 | Gv88 | CP012018 | 308 | 1971.CO1 | CP016858 | 309 | SVH7513 | CP029166 |
| 310 | HC1340 | CP012011 | 311 | FDAARGOS_2 | CP026964 | 312 | IT1-S | CP028468 |
| 313 | HC1335 | CP012012 | 314 | FDAARGOS_6 | CP026962 | 315 | IT4-R | CP028470 |
| 316 | MI | AP017320 | 317 | FDAARGOS_43 | CP026957 | 318 | MOK042 | CP029627 |
| 319 | TMUS2126 | AP014652 | 320 | FDAARGOS_48 | CP026953 | 321 | NRS143 | CP026071 |
| 322 | TMUS2134 | AP014653 | 323 | FORC_012 | CP010998 | 324 | DSM20231 | CP011526 |
| 325 | FDAARGOS_159 | CP014064 |  |  |  |  |  |  |

Table S2. Characteristics of 67 confirmed CRISPR loci of 52 *Staphylococci*

| Organism | Strain | Accession number | Length | Number of CRISPR | CRISPR length | Clonal Complex | MLST | *spa* | SCC*mec* |
| --- | --- | --- | --- | --- | --- | --- | --- | --- | --- |
| ***S. aureus*** | 104 | CP012409 | 2820837 | 1 | 191 | CC49 | ST49 | t208 | × |
| ***S. aureus*** | MOK063 | CP029629 | 2808798 | 1 | 191 | CC97 | ST97 | t359 | × |
| ***S. aureus*** | NCTC10344 | LS483324 | 2835275 | 1 | 191 | CC97 | ST97 | t359 | × |
| ***S. aureus*** | LGA251 | FR821779 | 2750834 | 1 | 196 | CC425 | ST425 | t6300 | ⅩⅠ |
| ***S. aureus*** | CA-347 | CP006044 | 2850503 | 1 | 199 | CC45 | ST45 | t004 | Ⅱ |
| ***S. aureus*** | AR466 | CP029080 | 2786146 | 1 | 199 | CC45 | ST45 | t282 | / |
| ***S. aureus*** | AR464 | CP029084 | 2793317 | 1 | 199 | CC45 | ST45 | t671 | Ⅳa |
| ***S. aureus*** | AR_0471 | CP029652 | 2785050 | 1 | 199 | CC45 | ST508 | t1203 | / |
| ***S. aureus*** | ST398 | AM990992 | 2872582 | 1 | 200 | CC398 | ST398 | t011 | Ⅴc |
| ***S. aureus*** | BA01611 | CP019945 | 2885865 | 1 | 200 | CC9 | ST9 | t899 | ⅩⅡ |
| ***S. aureus*** | RIVM1295 | CP013616 | 2777077 | 1 | 200 | CC398 | ST398 | t108 | Ⅴ |
| ***S. aureus*** | RIVM1607 | CP013619 | 2769573 | 1 | 200 | CC398 | ST398 | t011 | Ⅴc |
| ***S. aureus*** | RIVM3897 | CP013621 | 2894543 | 1 | 200 | CC398 | ST398 | t034 | Ⅴ |
| ***S. aureus*** | E154 | CP013218 | 2831848 | 1 | 200 | CC398 | ST398 | t034 | Ⅴc |
| ***S. aureus*** | 08S00974 | CP020019 | 2802697 | 1 | 200 | CC398 | ST398 | t011 | Ⅴc |
| ***S. aureus*** | 293G | CP019591 | 2745528 | 1 | 200 | CC398 | ST398 | t1451 | × |
| ***S. aureus*** | GD705 | CP019593 | 2832144 | 1 | 200 | CC398 | ST398 | t034 | × |
| ***S. aureus*** | GD1677 | CP019595 | 2813235 | 1 | 200 | CC398 | ST398 | t034 | × |
| ***S. aureus*** | GD1539 | CP019594 | 2819047 | 1 | 200 | CC398 | ST398 | t571 | × |
| ***S. aureus*** | ISU926 | CP017091 | 2833430 | 1 | 200 | CC398 | ST398 | t034 | Ⅴc |
| ***S. aureus*** | PTDrAP2 | CP029172 | 2902681 | 1 | 200 | CC398 | ST398 | t034 | Ⅴc |
| ***S. aureus*** | 5_3949 | LT992462 | 2959092 | 1 | 200 | CC398 | ST398 | t034 | Ⅴc |
| ***S. aureus*** | 8_LA_272 | LT992461 | 2891536 | 1 | 200 | CC398 | ST398 | t034 | Ⅴc |
| ***S. aureus*** | 2_LA_86 | LT992463 | 2857311 | 1 | 200 | CC398 | ST398 | t034 | Ⅴc |
| ***S. aureus*** | 16_LA_309 | LT992467 | 2866864 | 1 | 200 | CC398 | ST398 | t011 | Ⅴc |
| ***S. aureus*** | 12_LA_293 | LT992468 | 2910878 | 1 | 200 | CC398 | ST398 | t011 | Ⅴc |
| ***S. aureus*** | 17_LA_343 | LT992471 | 2922778 | 1 | 200 | CC398 | ST398 | t011 | Ⅳa |
| ***S. aureus*** | 10_5235 | LT992472 | 2811691 | 1 | 200 | CC398 | ST398 | t011 | Ⅴc |
| ***S. aureus*** | 19_LA_388 | LT992474 | 2862001 | 1 | 200 | CC398 | ST398 | t034 | Ⅴc |
| ***S. aureus*** | NCTC6136 | LS483311 | 2849927 | 1 | 200 | CC9 | ST9 | t4358 | × |
| ***S. aureus*** | JKD6159 | CP002114 | 2811435 | 1 | 201 | / | ST93 | t202 | Ⅳa |
| ***S. aureus*** | 55-99-44 | CP024998 | 2763876 | 1 | 204 | / | ST152 | t355 | ⅩⅢ |
| ***S. aureus*** | BB155 | LN854556 | 2778079 | 1 | 204 | / | ST152 | t355 | × |
| ***S. aureus*** | FDA209P | AP014942 | 2775733 | 1 | 247 | CC8 | ST464 | t3297 | × |
| ***S. aureus*** | NCTC3761 | LS483314 | 2774360 | 1 | 247 | / | / | t359 | × |
| ***S. aureus*** | ATCC6538 | CP020020 | 2772993 | 1 | 247 | CC8 | ST464 | t3297 | × |
| ***S. aureus*** | ST20130941 | CP012978 | 2678881 | 1 | 251 | CC15 | ST15 | t084 | × |
| ***S. aureus*** | 08BA02176 | CP003808 | 2782313 | 3 | 1108, 252, 200 | CC398 | ST398 | t034 | Ⅴ |
| ***S. aureus*** | JS395 | CP012756 | 2846866 | 2 | 463, 252 | CC395 | ST1093 | t359 | Ⅴ、Ⅳg |
| ***S. aureus*** | AR_0472 | CP029649 | 2834439 | 2 | 248, 891 | CC8 | ST72 | t148 | / |
| ***S. aureus*** | AR_0470 | CP029653 | 2963657 | 2 | 895, 252 | CC30 | ST30 | t017 | / |
| ***S. aureus*** | AR_0473 | CP029681 | 2834434 | 2 | 248, 891 | CC8 | ST72 | t148 | / |
| ***S. pseudintermedius*** | 063228 | NZ_CP015626 | 2766566 | 2 | 242, 252 |  |  |  |  |
| ***S. schleiferi*** | TSCC54 | NZ_AP014944 | 2528077 | 3 | 1179, 252, 1024 |  |  |  |  |
| ***S. equorum*** | KS1039 | NZ_CP013114 | 2822193 | 3 | 651, 612, 466 |  |  |  |  |
| ***S. argenteus*** | MSHR1132 | NC_016941 | 2762785 | 2 | 470, 326 |  |  |  |  |
| ***S. argenteus*** | XNO62 | NZ_CP023076 | 2744503 | 3 | 318, 402, 194 |  |  |  |  |
| ***S. argenteus*** | XNO106 | NZ_CP025023 | 2744502 | 3 | 318, 402, 194 |  |  |  |  |
| ***S. epidermidis*** | FDAARGOS_153 | NZ_CP014119 | 2497739 | 1 | 823 |  |  |  |  |
| ***S. epidermidis*** | RP62A | NC_002976 | 2616530 | 1 | 241 |  |  |  |  |
| ***S. lugdunensis*** | N920143 | NC_017353 | 2595888 | 1 | 323 |  |  |  |  |
| ***S. lugdunensis*** | HKU09-01 | NC_013893 | 2658366 | 1 | 466 |  |  |  |  |
| × represents MSSA and / represents undifferentiated type. | | | | | | | | | |

| Table S3. Characteristics of complete type III-A CRISPR-Cas system of 15 *Staphylococci*   \| **Organism** \| **Strain** \| **CRISPR Finder** \| \| \|  \| **CRISPRone** \| \| \| \| --- \| --- \| --- \| --- \| --- \| --- \| --- \| --- \| --- \| \| CRISPR array range \| Number of spacers \| Cas cluster \|  \| CRISPR array range \| Number of spacers \| Cas cluster \| \| ***S. aureus*** \| 08BA02176 \| 55513-56620 \| 15 \| Cas1_0_II,  Cas2_0_I-II-III, Cas10_0_IIIA, Csm2_0_IIIA, Csm3_0_IIIA, Csm4_0_IIIA, Csm5_0_IIIA, Csm6_0_IIIA, Cas6_0_I-III. \|  \| 55513-56620 \| 15 \| Cas1 universal,  Cas2 universal,  Cas10_III,  Csm2_III-A,  Csm3_III,  Csm3_III,  Csm3_III,  Csm6_III-A,  Cas6. \| \|  \|  \| 65343-65590 \| 3 \|  \|  \| 65343-65592 \| 3 \|  \| \| ***S. aureus*** \| JS395 \| 1407844-1408306 \| 6 \| Cas1_0_II,  Cas2_0_I-II-III, Cas10_0_IIIA, Csm2_0_IIIA, Csm3_0_IIIA, Csm4_0_IIIA, Csm5_0_IIIA, Csm6_0_IIIA, Cas6_0_I-III. \|  \| 1407844-1408304 \| 6 \| Cas1 universal,  Cas2 universal,  Cas10_III,  Csm2_III,  Csm3_III,  Csm3_III,  Csm3_III,  Csm6_III-A,  Cas6. \| \|  \|  \| 1417028-1417275 \| 3 \|  \|  \| 1417028-1417277 \| 3 \|  \| \| ***S. aureus*** \| AR_0472 \| 1725203- 1725450 \| 3 \| Cas6_0_I-III, Csm6_0_IIIA, Csm5_0_IIIA, Csm4_0_IIIA, Csm3_0_IIIA, Csm2_0_IIIA, Cas10_0_IIIA, Cas2_0_I-II-III, Cas1_0_II. \|  \| 1725199-1725450 \| 3 \| Cas6  Csm6_III-A,  Csm3_III,  Csm3_III,  Csm3_III,  Csm2_III-A  Cas10_III,  Cas2 universal,  Cas1 universal. \| \|  \|  \| 1734173- 1735063 \| 12 \|  \|  \| 1734173-1735063 \| 12 \|  \| \| ***S. aureus*** \| AR_0470 \| 61276-62170 \| 12 \| Cas1_0_II,  Cas2_0_I-II-III, Cas10_0_IIIA, Csm2_0_IIIA, Csm3_0_IIIA, Csm4_0_IIIA, Csm5_0_IIIA, Csm6_0_IIIA, Cas6_0_I-III. \|  \| 61276-62169 \| 12 \| Cas1 universal,  Cas2 universal,  Cas10_III,  Csm2_III-A,  Csm3_III,  Csm3_III,  Csm3_III,  Csm6_III-A,  Cas6. \| \|  \|  \| 70892-71139 \| 3 \|  \|  \| 70892-71141 \| 3 \|  \| \| ***S. aureus*** \| AR_0473 \| 2351273-2351520 \| 3 \| Cas6_0_I-III, Csm6_0_IIIA, Csm5_0_IIIA, Csm4_0_IIIA, Csm3_0_IIIA, Csm2_0_IIIA, Cas10_0_IIIA, Cas2_0_I-II-III, Cas1_0_II. \|  \| 2351269-2351520 \| 3 \| Cas6  Csm6_III-A,  Csm3_III,  Csm3_III,  Csm3_III,  Csm2_III-A  Cas10_III,  Cas2 universal,  Cas1 universal. \| \|  \|  \| 2360243-2361133 \| 12 \|  \|  \| 2360243-2361133 \| 12 \|  \| \| ***S. pseudintermedius*** \| 063228 \| 2664628-2664869 \| 3 \| Cas1_0_II,  Cas2_0_I-II-III, Cas10_0_IIIA, Csm2_0_IIIA, Csm3_0_IIIA, Csm4_0_IIIA, Csm5_0_IIIA, Csm6_0_IIIA, Cas6_0_I-III. \|  \| 2664628-2664874 \| 3 \| Cas1 universal,  Cas2 universal,  Cas10_III,  Csm2_III-A,  Csm3_III,  Csm3_III,  Csm3_III,  Csm6_III-A,  Cas6. \| \|  \|  \| 2673598-2673845 \| 3 \|  \|  \| 2673598-2673847 \| 3 \|  \| \| ***S. schleiferi*** \| TSCC54 \| 66853-68031 \| 16 \| Cas1_0_II,  Cas2_0_I-II-III, Cas10_0_IIIA, Csm2_0_IIIA, Csm3_0_IIIA, Csm4_0_IIIA, Csm5_0_IIIA, Csm6_0_IIIA, Cas6_0_I-III. \|  \| 66853-68031 \| 16 \| Cas1 universal,  Cas2 universal,  Cas10_III,  Csm2_III-A,  Csm3_III,  Csm3_III,  Csm3_III,  Csm6_III-A,  Cas6. \| \|  \|  \| 76758-77005 \| 3 \|  \|  \| 76758-77007 \| 3 \|  \| \|  \|  \| 1549038-1550061 \| 15 \| Cas2_0_I-II-III, Cas1_0_II,  Cas9_0_II. \|  \| 1549038-1550061 \| 15 \| Cas2_II-C, Cas1_II-C,  Cas9_II-C. \| \| ***S. equorum*** \| KS1039 \| 2723367-2723978 \| 8 \| Cas6_0_I-III, Csm6_0_IIIA, Csm5_0_IIIA, Csm4_0_IIIA, Csm3_0_IIIA, Csm2_0_IIIA, Cas10_0_IIIA, Cas2_0_I-II-III, Cas1_0_II. \|  \| 2723367-2723978 \| 8 \| Cas6  Csm6_III-A,  Csm3_III-D,  Csm3_III,  Csm3_III,  Csm2_III-A  Cas10_III,  Cas2 universal,  Cas1 universal. \| \|  \|  \| 2732728-2733193 \| 6 \|  \|  \| 2732728-2733193 \| 6 \|  \| \| ***S. argenteus*** \| MSHR1132 \| 61875-62344 \| 6 \| Cas1_0_II,  Cas2_0_I-II-III, Cas10_0_IIIA, Csm2_0_IIIA, Csm3_0_IIIA, Csm4_0_IIIA, Csm5_0_IIIA, Csm6_0_IIIA, Cas6_0_I-III. \|  \| 61875-62344 \| 6 \| Cas6  Csm6_III-A,  Csm3_III,  Csm3_III,  Csm3_III,  Csm2_III-A  Cas10_III,  Cas2 universal,  Cas1 universal. \| \|  \|  \| 71064-71389 \| 4 \|  \|  \| 71064-71389 \| 4 \|  \| \| ***S. argenteus*** \| XNO62 \| 37147-37464 \| 4 \| Cas1_0_II,  Cas2_0_I-II-III, Cas10_0_IIIA, Csm2_0_IIIA, Csm3_0_IIIA, Csm4_0_IIIA, Csm5_0_IIIA, Csm6_0_IIIA, Cas6_0_I-III. \|  \| 37147-37470 \| 4 \| Cas1 universal,  Cas2 universal,  Cas10_III,  Csm2_III-A,  Csm3_III,  Csm3_III,  Csm3_III,  Csm6_III-A,  Cas6. \| \|  \|  \| 46190-46591 \| 5 \|  \|  \| 46190-46591 \| 5 \|  \| \| ***S. argenteus*** \| XNO106 \| 37147-37464 \| 4 \| Cas1_0_II,  Cas2_0_I-II-III, Cas10_0_IIIA, Csm2_0_IIIA, Csm3_0_IIIA, Csm4_0_IIIA, Csm5_0_IIIA, Csm6_0_IIIA, Cas6_0_I-III. \|  \| 37147-37470 \| 4 \| Cas1 universal,  Cas2 universal,  Cas10_III,  Csm2_III-A,  Csm3_III,  Csm3_III,  Csm3_III,  Csm6_III-A,  Cas6. \| \|  \|  \| 46190-46591 \| 5 \|  \|  \| 46190-46591 \| 5 \|  \| \| ***S. epidermidis*** \| FDAARGOS_153 \| 968313-969135 \| 11 \| Cas6_0_I-III, Csm6_0_IIIA, Csm3_0_IIID, Csm4_0_IIIA, Csm3_0_IIIA, Csm2_0_IIIA, Cas10_0_IIIA, Cas2_0_I-II-III, Cas1_0_II. \|  \| 968313-969135 \| 11 \| Csx1_III  Cas6  Csm6_III-A,  Csm3_III-D,  Csm3_III,  Csm3_III,  Csm2_III-A  Cas10_III,  Cas2 universal,  Cas1 universal. \| \| ***S. epidermidis*** \| RP62A \| 2517620-2517868 \| 3 \| Cas6_0_I-III, Csm6_0_IIIA, Csm3_0_IIID, Csm4_0_IIIA, Csm3_0_IIIA, Csm2_0_IIIA, Cas10_0_IIIA, Cas2_0_I-II-III, Cas1_0_II. \|  \| 2517615-2517867 \| 3 \| Csx1_III  Cas6  Csm6_III-A,  Csm3_III,  Csm3_III,  Csm3_III,  Csm2_III-A  Cas10_III,  Cas2 universal,  Cas1 universal. \| \| ***S. lugdunensis*** \| N920143 \| 63455-63777 \| 4 \| Cas1_0_II,  Cas2_0_I-II-III, Cas10_0_IIIA, Csm2_0_IIIA, Csm3_0_IIIA, Csm4_0_IIIA, Csm3_0_IIID, Csm6_0_IIIA, Cas6_0_I-III. \|  \| 63455-63777 \| 4 \| Cas1 universal,  Cas2 universal,  Cas10_III,  Csm2_III-A,  Csm3_III,  Csm3_III,  Csm3_III-D,  Csm6_III-A,  Csm6_III-A,  Cas6. \| \| ***S. lugdunensis*** \| HKU09-01 \| 29464-29929 \| 6 \| Cas1_0_II,  Cas2_0_I-II-III, Cas10_0_IIIA, Csm2_0_IIIA, Csm3_0_IIIA, Csm4_0_IIIA, Csm3_0_IIID, Cas6_0_I-III. \|  \| 29464-29929 \| 6 \| Cas1 universal,  Cas2 universal,  Cas10_III,  Csm2_III-A,  Csm3_III,  Csm3_III,  Csm3_III-D,  Csm6_III-A,  Csm6_III-A,  Cas6. \|   Table S4. Sequences of 25 groups consensus direct repeat sequences (CDRs)   \| Group \| Strain \| CDRs sequence \| \| --- \| --- \| --- \| \| 1 \| TSCC54 \| GTTTCATTTATACCTAAAATTACAGAGTACTAAAAC \| \| 2 \| JKD6159 \| ATGCCATGTTACTTTGATGTGCTG \| \| 3 \| LGA251 \| TTCTCTATGTTGGGGCCCCGCCAA \| \| 4 \| ST398 \| TGCAAGTTGGCGGGGCCCCAACACAGAAGCT \| \| 5 \| 104 \| TCTGTGTTGGGGGCCTGCCAACT \| \| 6 \| FDA209P \| TGTTGGGGCCCCGCCAACTTGCA \| \| 7 \| ST20130941 \| TGCAAGTTGGCGGGGGCCCAACATAGA \| \| 8 \| AR_0473 \| CTCGTCCCCTCTTCTACGGGGTAGTTATCGAAT \| \| 9 \| KS1039 \| GTTCTCGTCCCCTATTCTTCGGGGTAGTTGTCGATC \| \| 10 \| AR_0473 \| ATTCTCGTCCCCTGTTATTCGGGGTAGTTATCGATC \| \| 11 \| KS1039 \| GTTCTCGTCCCCTCTTTTACGGGGTGGTTGTCGATT \| \| 12 \| FDAARGOS_153 \| GTTCTCGTCCCCTTTTCTTCGGGGTGGTTATCGATC \| \| 13 \| AR_0470 \| GATCGATAACTACCCCGAATAACAGGGGACGAGAATA \| \| 14 \| 08BA02176 \| GATCGATAACTACCCCGAATAACAGGGGACGAGAAT \| \| 15 \| 08BA02176 \| ATTCGATAACTACCCCGTAGAAGAGGGGACGAGAACT \| \| 16 \| 063228 \| GATCGATAACTACCCCGAATAACAGGGGAC \| \| 17 \| N920143 \| AATCAGAGAATACCCCGTATAAAAGGGGACGAGAAC \| \| 18 \| RP62A \| GATCGATACCCACCCCGAAGAAAAGGGGACGAGAAC \| \| 19 \| MSHR1132 \| ATTCGATAACTACCCCGAAGAAGAGGGGACGAGAACT \| \| 20 \| XNO62 \| TGATCGATAACTACCCCGAAGAATAGGGGAC \| \| 21 \| XNO106 \| TATTCGATAACTACCCCGAAGAAGAGGGGACGAGAAC \| \| 22 \| AR_0471 \| CCAGCTTCTGTGTTGGGGCCCCGCCAACTTG \| \| 23 \| XNO62 \| TGCAAGTTGGCGGGGCGCCAACACAGA \| \| 24 \| BA01611 \| TGTTGGGGCCCCACCCCAACTTGCA \| \| 25 \| CA-347 \| CAAGTTGGCGGGGCCCCAACACAGAAGCTGG \|   Table S5. The protospacer in phage and plasmid of spacer and their product | | |
| --- | --- | --- | --- | --- | --- | --- | --- | --- | --- | --- | --- | --- | --- | --- | --- | --- | --- | --- | --- | --- | --- | --- | --- | --- | --- | --- | --- | --- | --- | --- | --- | --- | --- | --- | --- | --- | --- | --- | --- | --- | --- | --- | --- | --- | --- | --- | --- | --- | --- | --- | --- | --- | --- | --- | --- | --- | --- | --- | --- | --- | --- | --- | --- | --- | --- | --- | --- | --- | --- | --- | --- | --- | --- | --- | --- | --- | --- | --- | --- | --- | --- | --- | --- | --- | --- | --- | --- | --- | --- | --- | --- | --- | --- | --- | --- | --- | --- | --- | --- | --- | --- | --- | --- | --- | --- | --- | --- | --- | --- | --- | --- | --- | --- | --- | --- | --- | --- | --- | --- | --- | --- | --- | --- | --- | --- | --- | --- | --- | --- | --- | --- | --- | --- | --- | --- | --- | --- | --- | --- | --- | --- | --- | --- | --- | --- | --- | --- | --- | --- | --- | --- | --- | --- | --- | --- | --- | --- | --- | --- | --- | --- | --- | --- | --- | --- | --- | --- | --- | --- | --- | --- | --- | --- | --- | --- | --- | --- | --- | --- | --- | --- | --- | --- | --- | --- | --- | --- | --- | --- | --- | --- | --- | --- | --- | --- | --- | --- | --- | --- | --- | --- | --- | --- | --- | --- | --- | --- | --- | --- | --- | --- | --- | --- | --- | --- | --- | --- | --- | --- | --- | --- | --- | --- | --- | --- | --- | --- | --- | --- | --- | --- | --- | --- | --- | --- | --- | --- | --- | --- | --- | --- | --- | --- | --- | --- | --- | --- | --- | --- | --- | --- | --- | --- | --- | --- | --- | --- | --- | --- | --- | --- | --- | --- | --- | --- | --- | --- | --- | --- | --- | --- | --- | --- | --- | --- | --- | --- | --- | --- | --- | --- | --- | --- | --- | --- | --- | --- | --- | --- | --- | --- | --- | --- | --- | --- | --- | --- | --- | --- | --- | --- | --- | --- | --- | --- | --- | --- | --- | --- | --- | --- | --- | --- | --- | --- | --- | --- | --- | --- | --- | --- | --- | --- | --- | --- | --- | --- | --- | --- | --- | --- | --- | --- | --- | --- | --- | --- | --- | --- |
| Spacers | Protospacers in phage and plasmid | Product |
| TTCTGTATTTCTATTAACCAATTCATAAGTGTCATTA (37) | phage CP220 | Possible phage major capsid protein |
| TGTTCCAATTAGCTAAAGATGTTATGGACGGCAAGA (36) | plasmid unnamed2 | terminase large subunit |
| GAGAACTTAATTGCATTATCAAATGTATATGCTGGATTCCA (41) | Bacteriophage 66 | ORF017 |
| GAGAACCCGAATTTTGATTCTTTGTTTGTAAATAATGCTC (40) | phage SAP-2, phage SCH111, phage PSa3, phage SLPW, phage SCH1, Bacteriophage 66, phage phi44AHJD, phage phiP68, phage vB_SauP_phiAGO1.9, phage vB_SauP_phiAGO1.3, phage BP39 | DNA binding protein |
| GAGAACCACGCTGTAGTGAAGTATAGAAACGGCATGAGTACAA (43) | phage phiSauS-IPLA88**;** phage StauST398-2, phage phi 13**;** Bacteriophage 52A**;** Bacteriophage 187**;** Bacteriophage 29**;** Bacteriophage 88**;** Bacteriophage X2**;** Bacteriophage 96**;** Bacteriophage 3A**;** Bacteriophage ROSA**;** Bacteriophage 42e**;** Bacteriophage 85**;** Bacteriophage 53**;** Bacteriophage 77**;** plasmid pVR-MSSA_02**;** Bacteriophage 47**;** phage Lorac**;** Bacteriophage 71**;** Bacteriophage 69**;** phage phiSauS-IPLA35**;** Bacteriophage 92**;** Bacteriophage 55 | gp9**;** phi PVL-like protein**;** ORF171**;** ORF105(ORF162)**;** ORF101(ORF191)**;** ORF188; ORF184**;** ORF092**;** ORF093**;** ORF193**;** ORF098(ORF175)**;** ORF082(ORF164)**;** ORF083(ORF176)**;** 77ORF102**;** DUF1270 family protein**;** ORF092(ORF161)**;** membrane protein**;** ORF105(ORF195)**;** ORF100**;** gp12**;** ORF086(ORF182)**;** ORF099 |
| CAAGGTCCATTAGTAGGTCGTGAAAATGAAGTTAA (35) | plasmid pMFPA43A1405A | phage tail protein |
| TAGTAAGTGATTTACATTATGACGGCATAGACGAACA (37) | phage vB_Sau_CG, phage vB_SauM_LM12, phage B1, phage JA1, phage phiIPLA-RODI, phage P4W, phage MSA6, phage Fi200W, phage 676Z, phage A3R, phage Staph1N, phage A5W, phage vB_Sau_S24, phage vB_Sau_Clo6**;** phage A5W, phage JA1, phage pSa-3, phage vB_Sau_S24, phage A3R, phage Staph1N, phage Staph1N**;** Bacteriophage G1**;** plasmid(NC_022111.1)**;** phage phiIPLA-C1C, phage vB_Sau_CG, phage vB_SauM_LM12, phage B1(MG656408), phage JA1, phage pSa-3, phage P4W, phage Fi200W, phage 676Z, phage A3R, phage A5W**;** Bacteriophage G1**;** plasmid pMR2**;** plasmid pBMB0233 | TreJ**;** terminal repeat encoded protein**;** ORF145; HlyC/CorC family transporter**;** TreK**;** ORF159**;** rnapol**;** HAMP domain-containing histidine kinase |
| ATCTGGAAGAGTGTTATCAATTTCAGTTACCTTAAAG (37) | phage Taffo16, phage Riley, phage Troll | helix-turn-helix DNA binding domain protein |
| AGTCAATATAAAGACAATACTTTTTACGCTTATATT (36) | phage phiIBB-SEP1, phage Quidividi, phage Twillingate | membrane protein |
| AAATTAATAAACTGAAAGAACCAAGAATTATTAT (34) | phage vB_SthS_VA214 | tail length tape-measure protein |
| AGAAGTATATGAAAATCATGAAAGTAATTTACTCAA (36) | phage vB_PreS_PR1; phage phiAS5 | minor tail protein**;** loader of DNA helicase |
| AATGAAATTTATCAAAACTATAGAAAACTTATTAG (35) | phage vB_StaM_SA2, phage vB_Sau_Clo6, phage vB_Sau_S24, phage MSA6, phage A3R, phage vB_SauM_LM12, phage vB_Sau_CG, phage B1, phage P4W, phage Fi200W, phage 676Z, phage Staph1N, phage Fi200W, phage A5W, phage phiIPLA-RODI**;** Bacteriophage G1 | TreP**;** ORF135 |
| GAATAAACTTGATTACAATATAGATTTAGAACATGA (36) | plasmid pDle1_3 | MBL fold metallo-hydrolase |
| TGGTTTAAGTTTGTCATTATAATCAATCCTTTTTCTT (37) | phage vB_Sau_S24, phage pSa-3 | terminal repeat-encoded protein |
| TGATTAAAACGGTTTGCTTTATTTGCATTTAAAATAG (37) | plasmid pBACSA01 | integration host factor subunit beta |
| GTTTTTCATAGTTAATCAATCCCTTTTCTTTTTT (34) | phage vB_SauM_LM12, phage pSa-3, phage P108, phage JD007, phage P4W, phage MSA6, phage 676Z, phage Staph1N, phage Fi200W, phage A3R, phage A5W**;** Bacteriophage G1; plasmid pMP1046A | Iro**;** ORF200**;** DUF1440 domain-containing protein |
| TTAAATCTTTGATTGCTCTTAGCTCTAGTTATGTAT (36) | Bacteriophage 88**;** Bacteriophage 92**;** Bacteriophage 55**;** Bacteriophage 3A**;** Bacteriophage 71**;** Bacteriophage 42e**;** phage phiSauS-IPLA35**;** Bacteriophage 52A**;** Bacteriophage 29**;** Bacteriophage 85**;** Bacteriophage 53**;** Bacteriophage 187**;** phage phiSa2wa_st1**;** plasmid pHD120112, plasmid pBT1850636 | ORF056**;** ORF059**;** ORF071**;** ORF071**;** ORF118**;** ORF081**;** gp29**;** ORF074**;** ORF060**;** ORF111**;** ORF078**;** ORF077**;** transcriptional activator RinB**;** IS4 family transposase |
| CACGCTGTAGTGAAGTATAGAAACGGCATGAGTACAAT (38) | phage StauST398-2, phage phi 13**;** phage phiSauS-IPLA88**;** Bacteriophage 52A**;** Bacteriophage 29**;** Bacteriophage 187**;** Bacteriophage X2**;** Bacteriophage 88**;** Bacteriophage 96**;** Bacteriophage 3A**;** Bacteriophage ROSA**;** Bacteriophage 42e**;** Bacteriophage 85**;** Bacteriophage 77**;** Bacteriophage 53**;** Bacteriophage 47**;** plasmid pVR-MSSA_02**;** phage Lorac**;** Bacteriophage 71**;** Bacteriophage 69**;** phage phiSauS-IPLA35**;** Bacteriophage 92**;** Bacteriophage 55 | phi PVL-like protein**;** gp9**;** ORF171**;** ORF191**;** ORF162**;** ORF184**;** ORF188**;** ORF092**;** ORF093**;** ORF193**;** ORF175**;** ORF164**;** 77ORF102**;** ORF176**;** ORF161**;** DUF1270 family protein**;** membrane protein**;** ORF195**;** ORF100**;** gp12**;** ORF182**;** ORF099 |
| TTTACTGTGTTTTTCATAATTAATCAATCCTTTA (34) | plasmid pHS18-1 | polysaccharide deacetylase |
| CTTCCGAATCCATTTCAGCGCAATAAACA (29) | phage SpT99F3, phage SA45ruMSSAST97, phage IME1346_01, phage SA537ruMSSAST97, phage SA7, phage JS01, phage tp310-3, phage phiPVL-CN125, phage 3 AJ-2017, phage SAP090B, phage tp310-1, phage phi 13 | terminase large subunit |
| ACTCACTTGTAAATTCCTCCACTTGCTCTA (30) | Bacteriophage 2638A | ORF019 |
| CTGGAATAACCACAAAGCCAGAGTCAGTTT (30) | phage phi575, phage SA7, phage SA45ruMSSAST97, phage IME1346_01, phage phi879, phage SA537ruMSSAST97, phage tp310-1, phage JS01, phage 3 AJ-2017, phage phiPVL-CN125, phage SAP090B; phage phi 13, phage tp310-3 | capsid protein; head protein |
| ACGTTAGATTTGCAGGTGTTAAGCACGGCT (30) | phage vB_SepS_SEP9, phage 6ec; phage IME1354_01 | preneck appendage protein; neck protein |
| ATGTTTTTTCATTAAAGCTACCAGTAATTC (30) | plasmid pAYP1020 | type IA DNA topoisomerase |
| AATTCGCCTTTATATTCAGGTTTCTTTTTT (30) | plasmid pEC1515-3, plasmid pEC974-3, plasmid pSalSendai, plasmid A(NZ_CP010181.1), plasmid A(NZ_CP010184.1), plasmid (NZ_CP011019.1)**;** plasmid pILYOP01 | antirestriction protein**;** class I SAM-dependent methyl transferase |
| TCTATAAGTTCATTAATTCCGATACCTAGATTATCT (36) | plasmid2 DNA, plasmid2 DNA | glyoxalase |
| TATACTATTTACATAATTTTTTATGTGTCTGTCTAC (36) | phage G | gp457(Bacillus virus G) |
| TAGAATGTTATTATCTAAGTGGTCGATGTATTCC (34) | phage P4W, phage MSA6, phage Fi200W, phage 676Z, phage Fi200W, phage A3R, phage Staph1N, phage A5W | TreQ |
| TCTGTAATGTATTCATTTAATGTAATCATAATTTTTTC (38) | plasmid pNDM1_010045, plasmid pOXA58_010030, plasmid pOXA58_100004, plasmid pOXA58_005078, plasmid pACI-df08, plasmid pXH901, plasmid IncAC-LS6**;** plasmid pACI-b25a, plasmid pNP7-1, plasmid pEC743_OXA48, plasmid pKp_Goe_070-2, plasmid unnamed1, plasmid pKp_Goe_208-2, plasmid pJEG011**;** plasmid pM131-2 | IS1 family transposase**;** IS1-like element ISPa14 family transposase**;** repeat_region(ISAba3 transposase, frameshift pseudogene) |
| TTTTCTTTAACTGTTTTTACTGCCCATTTAATAGT (35) | phage phiIPLA-C1C | TreK(potassium channel protein family) |
| AAGTTAACGGCATTACCTAATAAAAATATTTTAGG (35) | phage GRCS, phage BP39, phage SAP-2, phage phiP68, phage phi44AHJD, phage vB_SauP_phiAGO1.9, phage Idefix, phage St 134, phage Andhra**;** phage vB_Efae230P-4, phage vB_SauP_phiAGO1.3, phage SCH111, phage SCH1, phage PSa3, phage SLPW**;** phage SA4**;** Bacteriophage 66 | encapsidation protein**;** DNA packaging protein**;** neck appendage**;** ORF005 |
