# Supplementary material for: Characterization of 67 Confirmed Clustered Regularly Interspaced Short Palindromic Repeats Loci in 52 Strains of Staphylococci
Source: Front Microbiol. 2021 Oct 22;12:736565. doi: 10.3389/fmicb.2021.736565 (PMC8571024; doi:10.3389/fmicb.2021.736565)

Figure S1. The phylogenetic tree of *cas1*, *cas2* and the complete *cas* genes. “The complete *cas* genes” mean all *cas* genes in the complete CRISPR-Cas system in *Staphylococci*, except *cas* genes at orphan CRISPR locus.


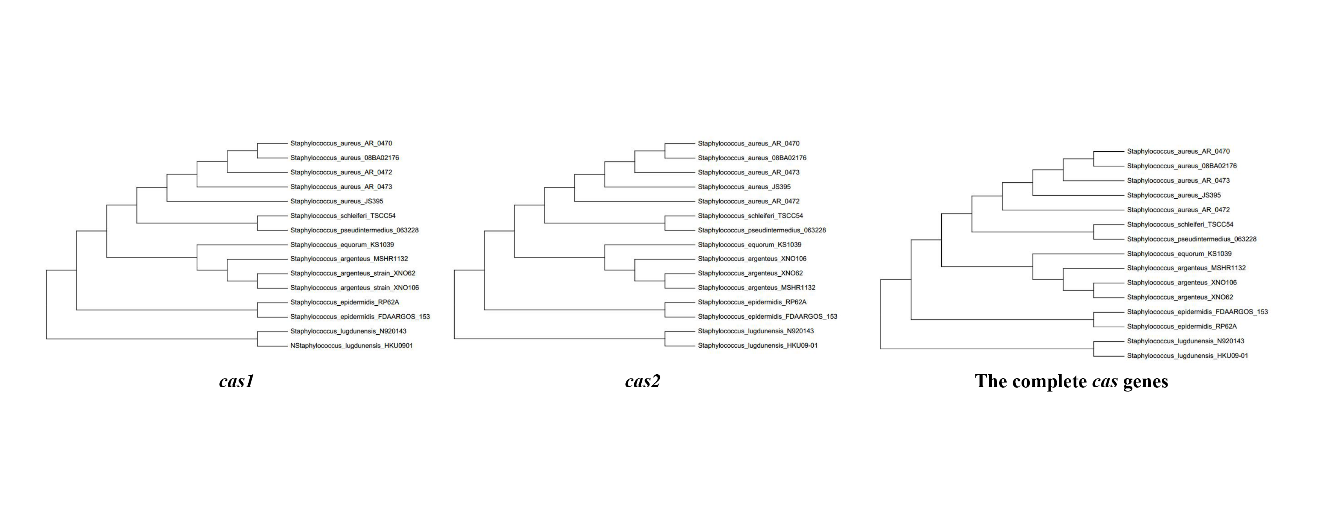

Supplement: Supplementary file 2 [file Data_Sheet_2.docx]
